# Exploring coping strategies and mental health support systems among female youth in the Northwest Territories using body mapping

**DOI:** 10.1080/22423982.2018.1466604

**Published:** 2018-04-26

**Authors:** Candice Lys

**Affiliations:** University of Toronto, Dalla Lana School of Public Health, Toronto, Ontario, Canada; Fostering Open eXpression among Youth (FOXY), Yellowknife, Northwest Territories, Canada

**Keywords:** Body mapping, Indigenous populations, intervention research, mental health, Northwest Territories, youth, qualitative methods

## Abstract

The mental health of young women in the Northwest Territories (NWT), Canada, is a critical public health concern; however, there is a dearth of research that examines how this population manages mental health challenges. This study explores the self-identified strategies that female youth in the NWT use to cope with mental health issues. The arts-based qualitative method of body mapping and a trauma-informed, strengths-based approach grounded in social ecological theory was used to collect data during in-depth semi-structured interviews. Forty-one participants (aged 13–17 years) attended FOXY body mapping workshops in six NWT communities in 2013 then completed interviews regarding the content of their body maps. Thematic analysis was used to identify five themes related to coping strategies: grounding via nature, strength through Indigenous cultures, connection with God and Christian beliefs, expression using the arts, and relationships with social supports. These results can be used to develop culturally relevant, strengths-based, trauma informed interventions that improve coping and resiliency among Northern youth.

## Introduction

Indigenous peoples in Canada experience a unique set of determinants that influence mental health [1], and these challenges are particularly evident in rural and remote regions, including the Northwest Territories (NWT). There is a growing burden of mental illness among people in rural communities and substantial disparities in health outcomes between those living in northern and southern Canada [2–4]. The rurality and geographic remoteness of the NWT influences availability of and access to mental healthcare provision [2,5]. Furthermore, the historical context of colonization and intergenerational trauma (often from residential schools) continues to have lasting impacts on the families of Northerners [6,7].

Youth who have experienced trauma may be more susceptible to mental health issues such as depression, self-harm, alcohol and other drug abuse and addiction, and anxiety [8]. Youth suicide rates are “alarmingly high” [6] in the Canadian Arctic, particularly among Indigenous youth, and in the NWT suicide rates are approximately 65% higher than the Canadian population [4]. The *NWT Addictions Survey* [9] found that 64% of youth in the NWT aged 15–24 years had participated in heavy drinking within the previous year, and 62% of this age group had engaged in harmful drinking patterns indicative of alcohol dependency. These mental health concerns are especially salient among young NWT women, who are disproportionately affected by trauma and violence [10]. Poor mental health during adolescence can have a significant long-term physical impact on young women and influence their development of self-esteem and ability to achieve healthy relationships with others.

Previous research has focused on the surveillance of mental health indicators among NWT youth such as rates of suicidal behaviour and suicide, self-reported mental health, and hospitalizations for mental illness diagnoses [4,6,11]. There is a dearth of current research regarding mental health coping strategies among adolescent women in the NWT; thus, the aim of this study is to explore the self-identified strategies that female youth in the NWT use to cope with mental health issues. Understanding the lived mental health experiences of young NWT women and their coping strategies is critical for informing the development of culturally relevant, strengths-based, and trauma informed interventions aimed to improve coping and resiliency that target Northern youth populations

## The FOXY intervention

**Figure 1.**
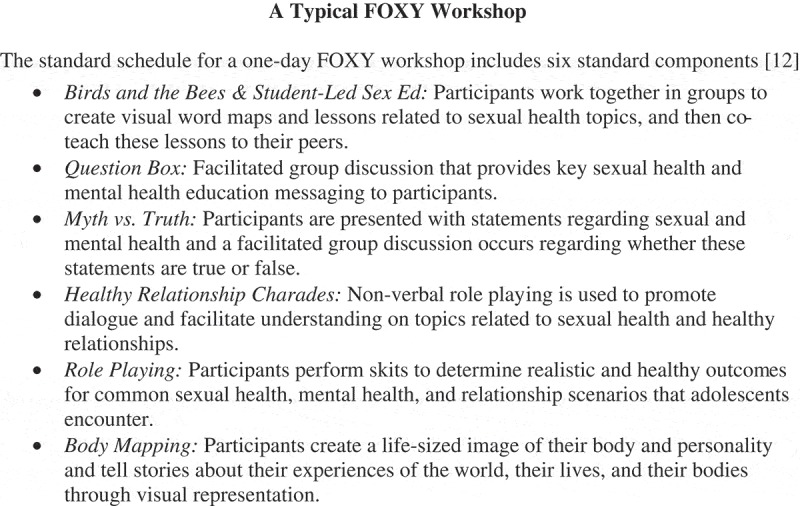
A typical FOXY workshop.

In response to the dearth of youth-driven public health interventions in the NWT, the researcher and other Northerners including youth developed an arts-based intervention called FOXY (Fostering Open eXpression among Youth) [12]. FOXY was developed for female-identifying (trans inclusive) youth and initially focused specifically on sexual health but evolved during its early implementation to include mental health as a core component. FOXY utilises a peer education model and employs adolescent Peer Leaders to co-facilitate strengths-based, trauma-informed workshops in schools with adult facilitators (See Figure 1. CALL OUT BOX). The FOXY intervention workshops focus on both visual and performance arts as a means to facilitate discussion and education about sexual and mental health. Mental health topics offered include educating about the impact of trauma; mental health issues common to Northern teenagers such as depression, suicide, self-harm, alcohol and other drug abuse; improving coping skills and help-seeking behaviours; and strengthening individual resiliency.

The researcher’s extensive personal and professional connection and involvement with the NWT as an Indigenous woman and life-long NWT resident enabled the development of this research project. The researcher actively participated as a co-facilitator of all FOXY workshops attended by study participants. This manuscript focuses on the body mapping component of the FOXY workshop intervention and specifically uses data from one-on-one interviews conducted to capture participants’ explanations of their body maps [13]. Body mapping is a guided exercise where participants create individual body maps on large sheets of paper using drawing, painting, or other media to visually represent aspects of an their lives, their bodies, and the world they live in [14]. Drawing symbols and images via body mapping is a strengths-based process that allows participants to tell their stories using a tool that helps to facilitate intergenerational dialogue (between youth and researcher), search for meaning within aspects of their lives, and engage in a strengths-based critical examination of their unique experiences that is not simply achieved through the qualitative interview process [14].

## Theoretical framework and methodological approach

This study was grounded in the social ecological theoretical framework. The social ecological framework emphasises that intrapersonal and interpersonal influences as well as environmental and policy contexts impact individual health behaviours and outcomes [15]. Body mapping was a guided activity that encouraged participants to reflect on their interpersonal relationships with partners, family members, peers, and others. As well, body mapping encouraged participants to depict characteristics of the intrapersonal self, internal resources, and personal resiliency [16]. Body mapping provided space for participants’ introspection regarding their body, health, and interpersonal relationships.

The social ecological framework guided the development of the body mapping intervention. A script with guided visualizations was developed, and creators added prompts to the visualizations to encourage participants to consider intrapersonal, interpersonal, environmental, and policy factors in their body map art, and then pilot tested it with Northern youth prior to use in the intervention. The social ecological framework also guided data analysis for this study, as lenses were applied for each of the social ecological levels during thematic analysis.

The experience of trauma is often a catalyst for mental health issues such as addictions, depression, and self-harm [8]. However, body mapping rejects the notion of “damage-centred” research that focuses on documenting the pain and brokenness of participants and pathologising their communities [17]. The body mapping exercise, and the larger FOXY workshop intervention, thus utilised a trauma-informed approach to support participants. This approach acknowledges that experiences of trauma are unique, that trauma responses are normal reactions to markedly distressing events that are outside of the usual realm of human experience, and a multitude of factors influence individual trauma stress responses, such as age, other past trauma, family dynamics, support systems, and many others [18]. Further, this approach recognises that individuals are not deficient because of their experiences and that there is no “correct” way to respond to trauma. Body mapping thus utilised this trauma-informed approach and a strengths-based lens to focus on the assets of the individual rather than on what the individual lacks, with the goal of self-empowerment. This process facilitated participants identifying and processing their own strengths, abilities, and internal and external resources [14]. This research project strived for a trauma-informed environment to support youth participants, which focused on attention to boundaries (between researcher and participant), language that communicated the values of empowerment, and creating safety for participants [19].

## Methods

To explore the intrapersonal and interpersonal contexts that influence the mental health of young women in the NWT and the strategies used to cope with mental health issues, two approaches were used for data collection: semi-structured interviews based on personal body maps and field notes.

### Recruitment & participants

From October to December 2013, the FOXY intervention was conducted at six schools across all five regions of the NWT (Beaufort Delta, Dehcho, North Slave, Sahtu, and South Slave), and these communities ranged in population from approximately 700 to 20,000 people. Purposive sampling [20] was used to recruit participants to attend the 1-day workshop. Community contacts (e.g. teachers, counsellors, administrators) invited students to consider attending the FOXY workshop due to their interest in the arts or because the community contact believed they would benefit from the workshop content. The FOXY intervention was delivered to all 57 female students who expressed interest in attending and all were invited to participate in the research study. Inclusion criteria for this study required that each participant: (a) identify as a woman (trans inclusive) between the ages of 13–18 years, (b) complete the FOXY intervention (including a body map), and (c) have parental consent to participate. 41 of the 57 FOXY workshop attendees opted to participate in this study by completing a body mapping interview; the remaining 16 chose not to due to disinterest in the research process, scheduling conflicts for interviews, and other unspecified reasons.

Participants ranged in age from 13 to 18 years old (*M* = 14.34) and all were currently in school, attending grades 7–12. All but four of the 41 youth participants had lived in the NWT for most, if not all, of their lives and the remaining four participants had lived in the NWT for a minimum of two years. 90% (*n* = 37) self-identified as Aboriginal or Indigenous (First Nations, Métis, or Inuit), 44% (*n* = 18) lived with both of their parents, 26% (*n* = 11) lived with one parent, 17% (*n* = 7) lived with other extended family members, and 12% (*n* = 5) lived with foster parents or in a group home at the time of data collection. 30 participants had never taken a FOXY workshop previously, seven had taken one FOXY workshop previously, and four had taken two or more FOXY workshops previously. None of the youth had participated in an interview regarding body mapping prior to this study.

Participants were engaged in the informed consent process prior to the start of the FOXY intervention. The researcher obtained written consent from all study participants, and participants were notified that all data would remain anonymous and confidential unless there was a reportable disclosure (e.g. child abuse). Parents/guardians were sent a letter during recruitment providing detail on the research project and a reverse consent form that asked them to return the signed form if they did not want their child/ward to participate in the study. This study received ethical approval from the University of Toronto HIV Community-based Research Ethics Board (Protocol #29038) and was also licensed by NWT Research License #15292.

### Data collection

Body map storytelling is a participatory qualitative research tool, and while the artwork can be data in themselves, these data were supplemented with individual interviews since the significance of the art can only be understood in relation to its creator’s reflexive account of their overall story and lived experiences [14]. Youth participants completed individual body maps during the FOXY workshop via a series of eight guided visualisations that were read to them by trained facilitators over 1.5 h. These visualisations were modified from the facilitator manuals by Solomon [21] and Gastaldo et al. [14] and added to during the initial piloting of FOXY workshops before the study commenced. Semi-structured interview guides were also pilot tested with six female youth respondents for comprehension, flow, order of questions, and utility of prompts and the interview guide was further refined prior to the study. Changes in interview protocols reflected a more in depth understanding of the research topic and setting, and member checking can increase validity of findings [22].

The researcher attended all workshops and recorded descriptive and reflective field notes during and after the FOXY intervention. Descriptive field notes included detailed accounts of what the researcher saw, heard, and experienced and reflective field notes further clarified and built on the descriptive field notes to reflect on the personal account of what the researcher learned during their observations [23]. Fieldwork also included active participation in FOXY intervention activities to build trust with participants, which led to informal conversations that provided the researcher with additional insights about the youth participants’ experiences.

After participating in the FOXY intervention, participants were asked to complete an in-depth interview that occurred in a private room in the school or at a location of the participant’s choice. Each participant’s body map was brought out during their individual interview and displayed so the participant could explain their artwork. The researcher asked participants a series of questions about their body map artwork using the semi-structured interview guide. The first question attempted to capture a personal narrative by asking the participant to explain the body map as they see it, as well as how the body map represents who they are. The personal narratives helped to contextualise and narrate the story conveyed in the body map, while the additional questions provided a “key” or detailed description of each body map symbol and element that granted access to the visual narrative and allowed for interpretation by the viewer [14]. The remaining questions asked participants to explain each part of their body map, including questions about where they came from and how they define themselves now, the challenges that they face as young women in the NWT, their internal and external resources, and their future aspirations, following the guided visualization script that facilitators read during the body mapping process. Interviews were audio-recorded and ranged from 27–67 min.

Body maps were photographed and labelled with each participants’ identification numbers after each interview, before being offered to the participants to take home. Participants were provided with a list of local, territorial, and national mental and sexual health resources. The researcher also connected with community counsellors during the recruitment process to ensure they would be available to participants if required and notified all participants of the counsellors available in their communities.

### Data analysis

All interviews were transcribed verbatim and identifying information was removed from transcripts. Interviews were analysed with ATLAS.ti Mac software (version 8.1.3) [24] using a thematic analysis process that was data driven (themes were not identified in advance of analysis) [25]. Data analysis consisted of multiple stages, including transcription, reading/familiarisation and re-reading transcripts, coding, searching for themes through the lens of different social ecological levels (e.g. intrapersonal, interpersonal, environmental, societal), producing a thematic map, naming and defining themes, and finalising analysis through writing [25]. Descriptive and reflective field notes were also read with individual transcripts to help immerse and familiarise the researcher with details of workshops and interviews. The analysis was an iterative process and the researcher revisited stages when necessary. Results are reported using the 32-item checklist of consolidated criteria for reporting qualitative research (COREQ) guidelines [26]. To ensure rigour in the process, interview data was triangulated with field notes to provide for a rich analysis, and interview transcripts were member checked with youth participants to ensure credibility and confirm accuracy [20].

## Results

Participants in this study self-identified that they experienced a breadth of mental health concerns. Depression, bipolar disorder, anxiety, alcohol and other drug abuse, self-harm, suicidal ideation and previous suicide attempts, and post-traumatic stress disorder (PTSD) were the most common concerns that participants discussed, often describing these issues as a result of trauma experienced through child abuse, family violence, sexual assault, bullying from peers, or intergenerational trauma resulting from family members who had attended residential schools.

Five prominent interrelated themes were identified through body map storytelling regarding the methods that female youth in the NWT used to cope with mental health issues at the intersecting intrapersonal (individual) and interpersonal (relationship) levels. These five themes included: grounding via nature, strength through Indigenous cultures, connection to God and religion, expression using the arts, and relationships with social supports.

### Grounding *via* nature

Symbols and drawings of nature in the North, such as the sun, water, butterflies, clouds, trees, animals (especially animals common in Northern Canada such as bears, foxes, ravens, and fish), the Inuksuk (rock formation used by people of the Arctic traditionally as markers for meat caches, directions, or warnings) (Participants drew and described their perception of what they referred to as an ‘Inuksuk,’ which was typically a figure that had a head, arms pointed outwards from the body, and two legs. Traditionally in Inuit culture, an Inuksuk is made without a head, arms, or legs (K. Mackay, personal communication, April 12, 2018).), fire, snow, and the aurora borealis were commonly included on participants’ body maps. These drawings were especially prominent in response to visualizations regarding where the individual felt they came from and the sources of their support systems. Many participants regarded nature as a grounding force that was a source of intrapersonal strength that made them feel more whole as individuals. One participant who struggled with alcohol and drug abuse, bipolar disorder, and the trauma of sexual assault also spoke of the importance of nature for her feeling grounded and safe:

*I love the outdoors, nature and stuff. I love animals and whenever I go hiking, I have a kayak that I go out on the lake with and stuff. I always feel so much peace outside, like there’s nothing that can hurt me and… I’ve always felt like nature has been a really big part of me. Especially because my family, they’re really outdoorsy, so that’s just kind of how I was born and raised*. [Participant 20]

The participant went on to describe being outdoors as a refuge from the negativity of society and a way to cope with feeling like she does not always fit in her community as a sexually diverse (queer) identified person with bipolar disorder:

*I don’t really agree with society that much. I’m kind of like the black sheep that everybody calls me. So I really want to get away from that. I don’t want to be part of all of society’s negativities*. [Participant 20]

Participants often discussed that being out on the land allowed them to disconnect from technology and the pressures of daily life, which they also found very grounding:

*I just like being out there and I feel like I’m not stuck in town or on mobile devices or on Facebook or stuff. I just like being on the land and having fun with people around me, instead of using Facebook to connect. I like being on the land*. [Participant 25]

Another participant discussed being out on the land as important for her self-identity and placing herself within her family structure. She described her drawings as:

*I drew things I did with all my family members when I was little. I would sing with my dad and not help cut wood, but sit there [laughs] while he cut wood outside, and go skidooing with my Grandpa*. [Participant 35]

### Strength through Indigenous cultures

**Figure 2.**
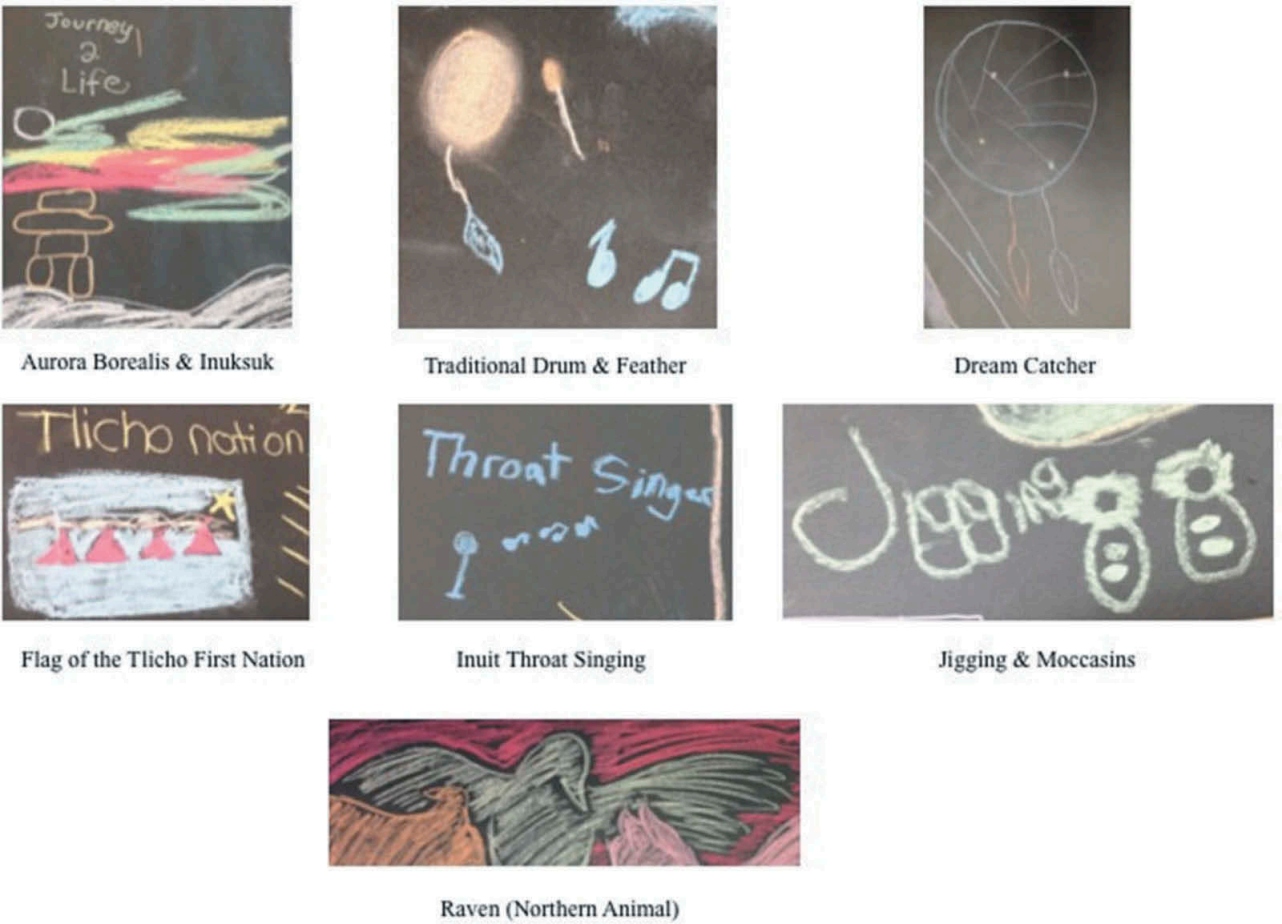
Examples of northern First Nations, Métis, and Inuit cultural symbols.

Many Indigenous participants identified connection with aspects of their cultures as protective factors that helped them to build and maintain their mental health. Symbols of connection to Northern First Nations, Métis, and Inuit cultures were drawn on body maps and examples are featured in Figure 2. These symbols included: animals and the aurora borealis found in Northern Canada; dream catchers, moccasins, and feathers that are important cultural symbols to many First Nations and Métis individuals; traditional hand drums; the Inuksuk used by the Inuit; the Métis sash; and flags representing First Nations communities. These symbols were typically drawn in the context of completing the guided visualisation that asks “where do you come from?” Participants often also described a connection to culture that was a core component of who they were currently and a resource for helping them to maintain strong, healthy lifestyles.

For many participants, traditional drumming, picking plants for use as traditional medicines, using traditional medicinal plants in ceremony, throat singing, jigging, hunting, trapping, and fishing, were important strength-building activities. One participant spoke of the importance of traditional hand drumming and how it gave her intrapersonal strength and self-confidence, helping her cope with depression:

*Since I’m M*é*tis, I drum with my aunties. We have a drumming group called [name withheld]. A memory I always think of is, whenever we go to the waterfalls and we sing there, we always offer tobacco to the earth and what not, and pray. So, that’s cool. Different things help me through these problems… Drumming has always made me feel more strong and more confident in myself. And just the singing and the songs, they’re just so powerful. And all of my aunties and my mom, they just sing so powerfully that just makes [me] feel good*. [Participant 15]

The opportunity to connect with their culture was made even more powerful when Elders were involved with the cultural activities. One participant explained the interpersonal relationship with her Grandma that she drew as her power symbol visualization:

*My Grandma, she teaches me about all the [traditional] medicines, and sometimes I go picking with her for [the traditional medicine] sweetgrass and stuff. Me having the opportunity is special. People don’t have that opportunity sometimes*. [Participant 25]

Another participant spoke in depth of her mother’s alcoholism and the impact of violence on her family and when asked if she had drawn anything to represent her hopes and dreams for the future, she responded: “*Only throat singing and [have a] happy family… I don’t want to be like my mom”* [Participant 39].

### Connection to God and Christian beliefs

Many participants expressed that they felt a connection to a Christian God and sometimes to religious beliefs or identified themselves as Christians. Several participants wrote words associated with Christianity such as “God” and “Jesus,” drew religious symbols such as a cross or a Bible, or wrote Bible verses on their body maps in the context of supports for when they were grappling with difficult emotions in reaction to situations at home (such as alcohol abuse among family members). When asked during their interviews, many participants explained that while they did not go to church they still related to core aspects of Christianity, such as Jesus, God, and the Bible. One participant explained that her belief in God helped to guide her when she was struggling emotionally: “I’m a believer in God and Jesus and I don’t go to church but I still pray in my Bible, or read a Bible verse when I need help” [Participant 1]. Another participant described her positive relationship with God when asked what she drew to represent an intrapersonal bodily cue that tells her she feels like she is in a healthy situation at home: “[I drew] the heart. When my heart says ‘yes,’ then you feel really happy and it sounds happier and you get really excited and energetic. And I put [the heart] with the ‘God’ too, because usually I think of God a lot when I’m happy” [Participant 2].

Several participants discussed their relationship with religion as giving them feelings of strength or safety. One participant described her religiosity as a source of strength and drew a cross with a Bible verse as her power symbol, describing it as: “I drew that God gives the biggest war to the strongest soldiers” [Participant 6]. Another participant also drew a cross, wrote ‘FEAR NOT ISAIAH 41:10’ and explained her personal power symbol as: “the Bible quote is the Fear Not Bible quote from Isaiah 41:10. It just makes me feel safe and like I can do anything… I don’t go to church but I’m Christian and I pray and stuff” [Participant 32]. Another participant explained that interpersonal relationships with other in her church youth group gave her a sense of safety: “I feel safe when I am with the youth group, because when we were together, it’s like we are all happy and no one is arguing, and it’s just fun and safe with them” [Participant 7].

For many participants, prayer was important for strengthening the connection to God, and one participant described her relationship with God through prayer as especially helpful for mitigating feelings of loneliness and coping with her diagnosis of bipolar disorder:

*I’ve always been [living] in a Christian household, but like a year or so ago I got into drinking and drugs and it was kind of brought on by some stuff that happened on a holiday before. So I kind of lost my relationship with [God] and then once I got put on some medicine for bipolar, everything kind of settled down and I became religious again. I think having God in my life, He is always something I can turn to because sometimes I feel kind of lonely and I need someone to talk to… I feel like I have God on my side when everyone else may not be. My mom always says I have something special with God so that’s pretty nice*. [Participant 20]

Another participant also explained her positive connection to God as: “If I was sad, I would just talk to [God]. If I wasn’t with Him, I’d be all sad and everything” [Participant 7].

### Expression using the arts

Several participants used drawing to express themselves and as a therapeutic tool to process their feelings and recognise their intrapersonal strengths, which can help individuals struggling with their mental health to balance their emotions and ground themselves. One participant spoke of the value of creating art for her:

*I express myself through art and writing and it’s just something that’s super important to me. If I ever needed something to get my mind off things, I would go straight to art and go draw this crazy drawing with black and purple and all the emotion in it. And I used to write poetry and I still do sometimes. I just find art really important. I really like how we did body mapping in the FOXY workshop, because it does get you to understand yourself better and it makes you realize that it’s not all bad and there’s a lot of good in you too*. [Participant 19]

Another participant who experienced anxiety often due to her parents’ fighting, explained that art was important to her because it made her feel empowered and confident and so she included art as part of her personal power symbol visualization:

*When I found spoken word poetry, I was really happy and I just like love it and I really connect to it. And last year I actually won a spoken word poetry contest. It was a junior high poetry contest and we had to read it in front of four judges and our peers. It was really nerve wracking but I won. [Poetry] just like keeps me going and makes me feel good about myself and feel like I can do or be anything I want and it’s okay*. [Participant 32]

Symbols of music were noticeable on many of the body maps. One participant described her connection with music as an intrapersonal coping mechanism to help her through stress and difficulties expressing emotions:

*When I was going through really rough times, I always looked to music as a help. Now I just find it still does [help], but in a totally different way. I used to blast my Black Veil Brides [songs] and my crazy music, and that made me less stressed out and less angry because usually when I get mad and stuff I kind of bottle it up inside. And so music kind of expresses it for me. I don’t like singing in front of people, so listening to other people sing is nice*. [Participant 20]

When asked to explain why she included music as an obvious key component of her body map, another participant responded:

*The bottom [of the body map] is music. I feel like I come from music because I really like music. I have a whole bunch of songs on my iPod. It helps me cope with stress a lot.* [Participant 29]

Several participants also wrote song lyrics on their body maps and spoke about their connection with music by their favourite bands, or their ability to play instruments.

### Relationships and social supports

Participants identified several interpersonal relationships as key to supporting their mental health. Some participants felt that their parents were strong supports for them, helping them to make healthy decisions. For those who expressed that a parent was a positive support, most participants identified closer relationships with their mothers than fathers. One participant drew a vulva on her body map during the visualization to represent her mom, and explained the drawing accordingly:

*My mom is different from all the moms here [in my community]… She has this attitude… like you can do anything if you do it proper, I guess. If you go to school you can do all this. She’s so funny, and other moms here are so… I don’t know, they drink and stuff and do drugs. My mom, she’s not into that. She’s completely different from other moms*. [Participant 24]

However, for many participants, biological parents were not always the strongest support systems. Several participants described complicated interpersonal relationships with their parents, often due to their parents’ problem drinking and the consequences of this drinking. As one participant described:

*When I was a kid, my parents were always there for me. My mom loves me, and my dad kind of [loves me] I think, because he’s not my real dad. One thing I don’t like about them is they drink a lot… That’s the reason why I moved in with my auntie. I’m going to school more often, doing my homework and stuff*. [Participant 28]

Another participant also found support from her aunt when her mom was drinking: “My auntie [name withheld] helps me, because my mom kind of drinks all the time and [auntie] told me I could go over to her house anytime that I want” [Participant 38].

For many participants, aunts and uncles, godparents, grandparents, and cousins were common family supports who were discussed at length. One participant wrote “Grandma” in the support shadow on her body map that detailed her support system, and explained her drawing accordingly:

*Anything in my life that I do, she’s always there to support me, and she’ll help out in the best way that she can, whether it’s research for projects I’m doing, or help with money or help with just emotional support. [When there is] drama with friends, she’s there saying that I’m better than that and I can get through anything, and she’s always just there for me… That’s why I put her in my support shadow, cuz like with anything, she’s there. She’s almost like a mom basically. Like, a second mom*. [Participant 35]

Another participant explained that her aunt was key to her resisting addictions and keeping on a good life path: “[My auntie] gives me a lot of stuff and she tells me that smoking is really bad. She keeps me from staying home from school. She keeps me active because she paid my way into hockey” [Participant 8].

Generally, participants had stronger bonds with female relatives compared with male relatives. A participant described her sister as an important role model who helped her to avoid the challenging behaviours associated with drinking alcohol that were common among her other family members. The close relationship with her sister helped to her to build resistance to using substances:

*My older sister supports me not to do drugs or smoke [tobacco] or drink. Right now she smokes and drinks, but she is trying to quit. She shows me how hard it is to quit if I ever started. On Facebook, it said that she wished she would move back into my Grandma’s house so she could be closer to us. She said she would protect us and keep us from drinking. Sometimes she takes us to our other Grandma’s house when my uncle is drinking. So she told my Grandma that we are going to my other Grandma’s. Before we do that, we check and see if anyone is drinking there. And if everyone is sober, then we go and stay there for a night, then in the morning we call my other Grandma’s house to see if my uncle is still drunk. If he is, then we would stay there for the day. If he sobers up during the day then we could go home*. [Participant 3]

Another participant also described her sister as critical for keeping her away from substance use:

*She always tells me, I hope you don’t turn to drinking and I’m like, no, I won’t. And then sometimes she’ll say ‘I’ll pick you up for lunch and we’ll talk.’ She says to spend time with family, because she realizes too that if I don’t spend time with family then I’m getting in trouble*. [Participant 6]

For many participants in this study, living with family members other than their mother or father (e.g. grandparents, godparents, aunts/uncles, adoptive parents, and foster parents) was common. One participant discussed living with her grandmother, who she referred to as her “Mama:” “In grade 7, me and my mom were like in our bad stages and stuff so I moved to [home community] with my Mama. She helped me a lot and she like told me everything was going to be okay. She helped me be stronger” [Participant 13]. A participant currently living in foster care also described her foster parent as a strong support for their mental health: “[Name withheld] is just my foster mom, but you can say she is my best friend” [Participant 40].

Only 1 of the 41 participants mentioned a mental health provider (a psychiatrist) that was outside of the school system as a strong support for their mental health issues. Frequently, participants discussed counsellors within their school as helpful to speak with about their problems. For example, one participant stated: “Whenever I have a bad day, I just go up to [the school counsellor] and she helps me. After I’m done talking with her, I am in a good mood” [Participant 1]. Another participant discussed the support she felt from her school counsellor to stop her self-harming behaviour:

*The counselor that I had here [at school], I guess you could say that we are kind of friends. I really liked her. She was really nice. She was supportive. She really helped me. I kind of just went to her when something went wrong. She’d take me during one of my classes and if I was sad that day, she would give me something to do. I had to do these self-injury things, like saying things that you didn’t like about yourself and things that you loved about yourself and at the time, things I loved about myself were very low and everything else was about the things I didn’t like about myself. She helped me change that because I’m more appreciative of how I look now. She was kind of my biggest support along with my friends*. [Participant 22]

Participants who attended the same school often identified the same teacher as an ally to young people. One participant identified her teacher as the only social support in her life: “I told her I was feeling really stressed lately and she told my other teachers about homework and stuff. There’s not really anybody else on the body map who is a support” [Participant 25].

Peers were significant influences on the mental health of participants and their ability to cope with difficult situations. One participant summarised her interpersonal relationships with her friends as: “they make me feel really good about myself. They make me laugh, and whenever like I’m sad or something, they are always there for me” [Participant 13]. Another participant described her friends as supportive of her decisions: “They don’t pressure me. They’ll be like um, this is your choice, I’m not going to influence you in your choices. And I think my choices are pretty good” [Participant 19]. One participant spoke about the bond that she had with her friend who supported her when she needed a place to stay when family members were on a drinking binge:

*One of my friends… now if I ever need something or need someone to go cry on or whatever, she’ll always be there. She used to let me sleep on her couch when I didn’t have somewhere to stay, but now it’s pretty different and my life has changed a lot. I’ve grown up a lot in my own beliefs and personality and stuff. It’s pretty different now but I still know that they’ll be there if I need them*. [Participant 20]

When asked about a lock she drew on her body map, another participant spoke about her experiences being bullied and how her friends made her feel protected from bullies:

*The lock is about safety. Since I started talking to all these other [friends] and they’re telling me that they’re always there or whatever and I’m not alone, I’m starting to feel more safe around school and out in public and everything*. [Participant 5]

## Discussion

This study provided a supportive, trauma-informed environment for young Northern women to share their experiences and explore the intrapersonal and interpersonal contexts (within a social ecological framework) that influence their mental health and the strengths-based strategies they use to cope with mental health issues. Nature was used to develop intrapersonal grounding and resiliency, and participants perceived that being out on the land was a desired break from technology and the negativity they felt in their daily lives. Nearly all participants (90%) self-identified as Aboriginal or Indigenous, and this intrapersonal bond with their cultures strengthened resiliency and helped participants cope directly with their mental health issues. Reclamation of Indigenous practices that were lost during historical periods of colonization, such as traditional activities on the land, are fundamentally acts of healing from trauma for both individuals and their communities, strengthening both intrapersonal notions of Indigenous identity and interpersonal relationships among community members [7]. Felt connections to God and, for some, religious beliefs (particularly Christianity) were important sources of intrapersonal strength or perceived safety for many participants, and prayer was described as helpful in decision-making in complex situations. However, participants in this study did not indicate that church attendance was a coping strategy. This finding is in contrast to other studies with Indigenous youth in the Canadian North that have found that church attendance is a protective factor for mental health not due to religious beliefs or practices, but rather a result of the social engagement with community members and strengthened interpersonal relationships within communities that occur from attending church [6,27]. The arts were useful for building intrapersonal strengths such as emotional processing of feelings and self-confidence, and acted as a catalyst for coping during difficult moments. Finally, participants identified interpersonal relationships with social supports as key to maintaining their mental health, including with their parents, other family members and foster/adoptive parents (particularly other females), counsellors, specific teachers, and peers.

A key finding in this study was that the vast majority of participants did not identify relationships with mental healthcare providers outside of their schools as mental health supports. This finding, along with the literature [3,28–31], suggest that Indigenous and Northern youth currently utilise a range of supports outside the formal healthcare system to manage their mental health. Previous studies have found that health services are often difficult to access for Northerners and Indigenous peoples due to diverse barriers that often exist within the environmental or policy contexts of the social ecological framework, such as staffing shortages and high staff turnover [5], geography [5,32], structural racism in the healthcare system [33], and the unavailability of consistent culturally relevant and safe care [5,34]. Findings from this study thus corroborate past research that describe how Indigenous and Northern Canadian youth rely on intrapersonal and interpersonal supports that are not part of the healthcare system when addressing mental health challenges. These supports include connection to culture, traditional customs, and practices [30,35,36], being out on the land and connecting to the environment [7,29], using the arts to build personal resiliency and strengthen peer relationships [28,37], leaning on family and other individuals [29,31], and practising their religious faith [3].

Strengths-based mental health interventions previously implemented in the North reported that empowering participants to express their own interpretations of their lived experiences through participatory visual arts methodologies can promote mental health and wellness [37]. However, the method of body mapping has not previously been used in mental health intervention research with youth in Northern Canada, and this study provides key findings on how this method helps to elicit the strengths and coping strategies of young NWT women. Specifically, body mapping helped youth to identify and depict individual power symbols that they can refer to when needed. Further, body mapping facilitated participants identifying intrapersonal resources that they can access within themselves, their interpersonal relationships, and their communities. Strengths-based approaches to resource identification with youth can strengthen protective factors, such as adaptive coping strategies, for enhancing the mental health of Northern Indigenous youth [3].

## Conclusion and implications for practice

While this study focused on the intrapersonal and interpersonal factors that influence coping strategies, findings can best advance change by also engaging environmental and policy contexts to help strengthen mental health for young women in the NWT, suggesting the importance of a social ecological perspective [38]. Many participants acted as interpersonal supports for other young people, providing safety and shelter for friends in need, especially when family members were using alcohol or during periods of family violence. This highlights the pivotal role of interpersonal resources in advancing mental health in this context.

Maslow identifies safety as an basic human need that must be met before other needs can be achieved, such as psychological needs and self-actualisation [39,40]. Thus, individuals who work with youth should ensure that mental health interventions are trauma-informed and give attention to both developing external safety (actions that youth can take to effect change/safety in their surroundings) and internal safety (intrapersonal coping skills) [18]. Youth may benefit from multi-level mental health interventions that focus on strengthening intrapersonal coping skills, and may include techniques of self-soothing, mindfulness, breathing, body awareness, and grounding [18,41].

Trauma-informed training [18] for informal healthcare providers (such as teachers) who can provide basic support in communities that lack reliable and consistent mental healthcare provision may be a critical resource for improving youth mental health in the North. NWT teachers often provide a vital support role to students regarding mental health. Future research should focus on building the skills of Northern teachers as informal mental health providers, and providing support to help them identify and process vicarious trauma that can result from empathic engagement with traumatized youth. Policy makers should focus on policies that support healthcare providers to remove barriers (such as concerns about privacy and confidentiality) that youth face to accessing the mental health system and services. Community-based interventions should focus on increasing safety for Northern youth and their families, for instance by supporting the healing of intergenerational trauma and its effects (addictions, violence, and abuse) among community members [42]. Finally, promising future mental health interventions that target Northern youth should consider: (1) encouraging youth to recognise and access intrapersonal, interpersonal, and cultural strengths and resources; (2) building on personal resiliency and adaptive coping strategies among youth; and, (3) working with youth to identify and connect with individuals in their communities who they feel are positive, healthy social supports.
